# Supplementary material for: In Vivo Inhibition of TRPC6 by SH045 Attenuates Renal Fibrosis in a New Zealand Obese (NZO) Mouse Model of Metabolic Syndrome
Source: Int J Mol Sci. 2022 Jun 20;23(12):6870. doi: 10.3390/ijms23126870 (PMC9224794; doi:10.3390/ijms23126870)
Supplement: Supplementary file 1 [file ijms-23-06870-s001.zip › ijms-1663959-supplementary.pdf]

International Journal of *Molecular Sciences*

MDPI

In vivo inhibition of TRPC6 by SH045 attenuates renal fibrosis in the New Zealand obese (NZO) mouse model of metabolic syndrome

Zhihuang Zheng[1,2,3], Yao Xu[4], Ute Krügel[5], Michael Schaefer[5], Tilman Grune[6,7], Bernd Nürnberg[8], May-Britt Köhler[2], Maik Gollasch[1,4*], Dmitry Tsvetkov[4*] and Lajos Markó[2,7,9,10*]

1 Department of Nephrology/Intensive Care, Charité - Universitätsmedizin, Corporate Member of Freie Universität Berlin, Humboldt-Universität zu Berlin, Berlin, Germany.
2 Experimental and Clinical Research Center, a cooperation of Charité - Universitätsmedizin Berlin and Max Delbrück Center for Molecular Medicine, Berlin, Germany.
3 Department of Nephrology, Shanghai General Hospital, Shanghai Jiaotong University School of Medicine, Shanghai, China.
4 Department of Geriatrics, University of Greifswald, University District Hospital Wolgast, Greifswald, Germany.
5 Rudolf Boehm Institute for Pharmacology and Toxicology, Leipzig University, Leipzig, Germany.
6 Department of Molecular Toxicology, German Institute of Human Nutrition Potsdam-Rehbruecke (DIfE), Nuthetal, Germany.
7 DZHK (German Centre for Cardiovascular Research), partner site Berlin, Germany.
8 Department of Pharmacology, Experimental Therapy and Toxicology and Interfaculty Center of Pharmacogenomics and Drug Research, University of Tübingen, Tübingen, Germany.
9 Berlin Institute of Health at Charité - Universitätsmedizin Berlin, Berlin, Germany.
10 Charité - Universitätsmedizin Berlin, Corporate Member of Freie Universität Berlin and Humboldt-Universität zu Berlin, Berlin, Germany.

*The corresponding authors: M.G., maik.gollasch@med.uni-greifswald.de ; D.T., dmitry.tsvetkov@med.uni-greifswald.de; L.M., lajos.marko@charite.de .

Figure S1

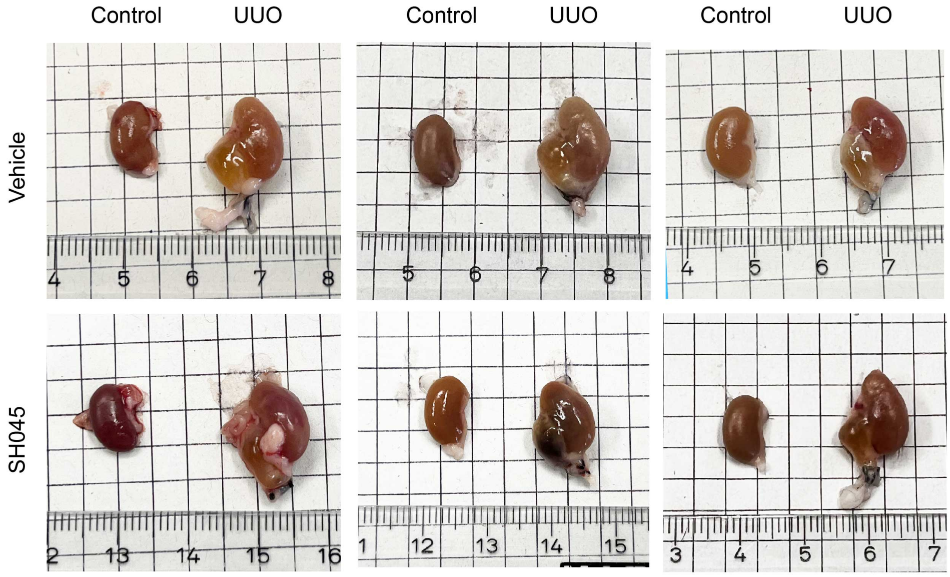

Figure S1. Macroscopic comparison of the kidneys. Images of control kidneys (left) and UUO kidneys (right). The UUO kidney increased in size (hydronephrosis) compared to control kidney. Scale bar: 1cm.

Figure S2

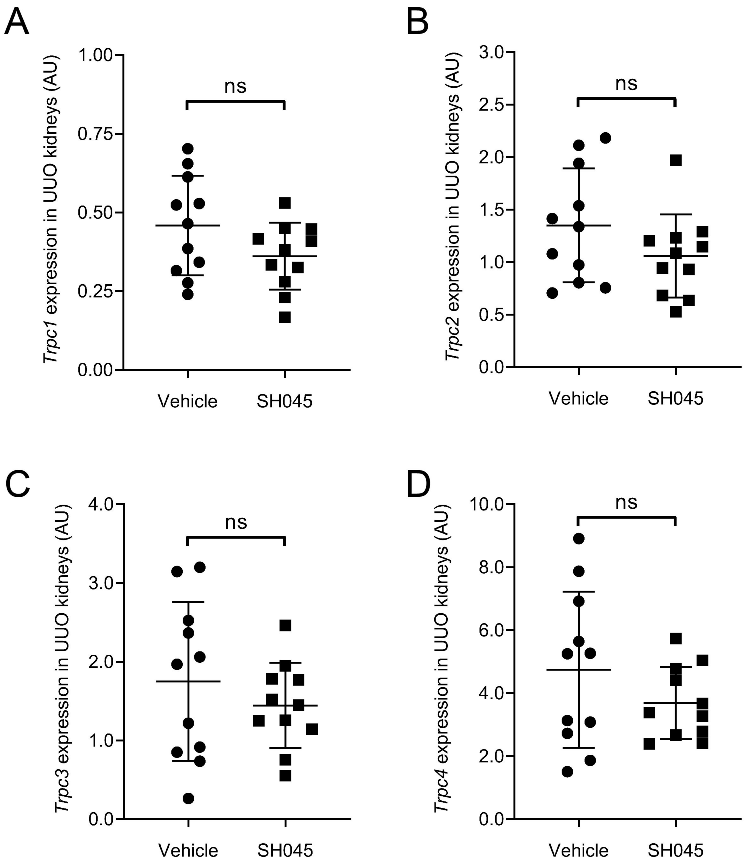

Figure S2. The impact of SH045 on renal *Trpc* gene expression in UUO kidneys. (A) Renal mRNA levels of *Trpc1*, (B) *Trpc2*, (C) *Trpc3*, and (D) *Trpc4* (n=11 each). Data expressed as means ± SD. Two-way ANOVA followed by Sidak's multiple comparisons post hoc test. ns, not statistically significant. AU, arbitrary units.

Figure S3

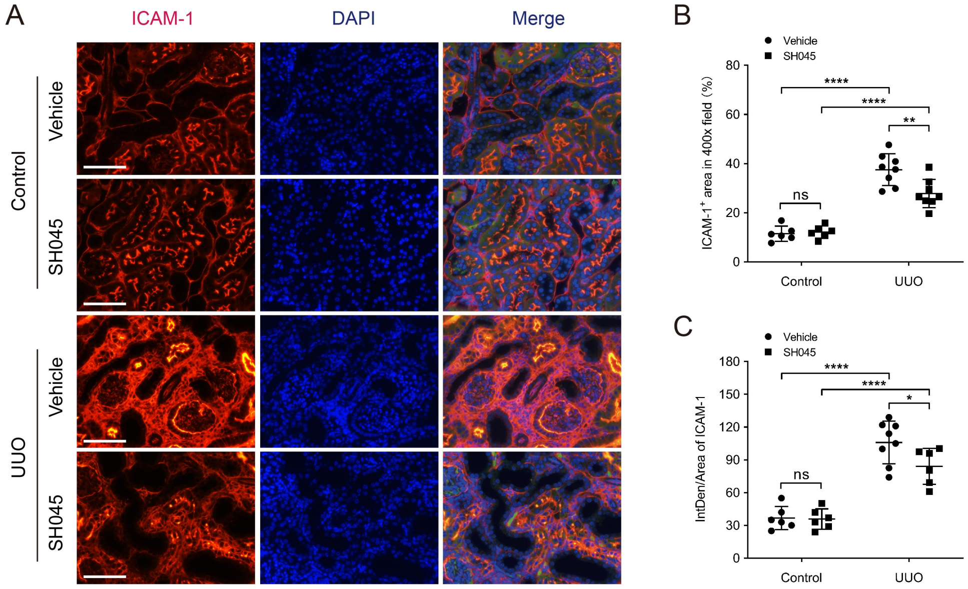

Figure S3. SH045 impact on renal ICAM-1 expression after UUO. (A) Renal immunofluorescence of ICAM-1. (B) Quantification of renal ICAM-1 expression in area proportion (Control n=6 each, UUO n=8 each, respectively). (C) Quantification of renal ICAM-1 expression in mean density (Control n=6 each, UUO n=8 each, respectively). Data expressed as means ± SD. Two-way ANOVA followed by Sidak's multiple comparisons post hoc test. *$p<0.05$, **$p<0.01$, and ****$p<0.0001$ defined as significant. ns, not statistically significant. IntDen, integrated density.

Table S1

Table S1. SH045 impact on blood parameters in NZO-UUO mice. Blood parameters after 7-days UUO (n=9, 8 for UUO vehicle and UUO SH045, respectively). Data expressed as means ± SD. Two-tailed unpaired t-test. n.a. = not applicable.

		UUO+Vehicle		UUO+SH045		*p*-value
Serum parameter	Unit	Mean	SD	Mean	SD	t-test
Sodium	mmol/L	147.30	1.25	146.88	2.17	0.608
Potassium	mmol/L	4.57	0.30	4.49	0.78	0.760
Chloride	mmol/L	109.40	1.17	110.75	1.49	0.047
Ionized Calcium	mmol/L	1.38	0.20	1.33	0.05	0.563
Total Carbon Dioxide	mmol/L	27.20	1.40	26.44	1.81	0.320
Glucose	mg/dL	240.10	43.83	229.56	62.19	0.672
Hematocrit	% PCV	40.10	4.43	41.63	3.62	0.444
Hemoglobin	g/dL	13.63	1.51	14.16	1.22	0.432
Anion Gap	mmol/L	16.40	2.27	15.25	1.28	0.221

Table S2

Table S2. Primers used in qRT-PCR experiments.

Primers	Forward	Reverse
Havcrt (KIM-1)	5'-CTGGAGTAATCACACTGAAGCAATG-3'	5'-GATGCCAACATAGAAGCCCTTAGT-3'
Lcn2 (NGAL)	5'-ATGTCACCTCCATCCTGGTCAG-3'	5'-GCCACTTGCACATTGTAGCTCTG-3'
Trpc1	5'-CATCTAGCGATGAGCCTCTTGAC-3'	5'-CCAAACCGTGTTCAGGAACTGC-3'
Trpc2	5'-AAGACTGCGGTTGGCTGTCAAC-3'	5'-GGCAACAAAGAGCTTCCAGATGG-3'
Trpc3	5'-ATTCCTCGCCATCGGCTATTGG-3'	5'-GAGGCGTTGAATACAAGCAGACC-3'
Trpc4	5'-CTTGAACAGGCAAGGTCCACCA-3'	5'-TGTAATCCTGGAGTCCGCCATC-3'
Trpc6	5'-GACCGTTCATGAAGTTTGTAGCAC-3'	5'-AGTATTCTTTGGGGCCTTGAGTCC-3'
Col1α1	5'-CATGTTCAGCTTTGTGGACCT-3'	5'-GCAGCTGACTTCAGGGATGT-3'
Col3α1	5'-CTCACCCTTCTTCATCCCACTCTTA-3'	5'-ACATGGTTCTGGCTTCCAGACAT-3'
Col4α1	5'-TTAAAGGACTCCAGGGACCAC-3'	5'-CCCACTGAGCCTGTCACAC-3'
Acta2 (α-SMA)	5'-ACTGGGACGACATGGAAAAG-3'	5'-CATCTCCAGAGTCCAGCACA-3'
Ccn2 (CTGF)	5'-TGCGAAGCTGACCTGGAGGAAA-3'	5'-CCGCAGAACTTAGCCCTGTATG-3'
Fn1 (Fibronectin)	5'-CCCTATCTCTGATACCGTTGTCC-3'	5'-TGCCGCAACTACTGTGATTCGG-3'
Ccl2	5'-GCTACAAGAGGATCACCAGCAG-3'	5'-GTCTGGACCCATTCCTTCTTGG-3'
Ccl5	5'-CCTGCTGCTTTGCCTACCTCTC-3'	5'-ACACACTTGGCGGTTCCTTCGA-3'
Ccr2	5'-GCTGTGTTTGCCTCTCTACCAG-3'	5'-CAAGTAGAGGCAGGATCAGGCT-3'
Cxcl1	5'-TCCAGAGCTTGAAGGTGTTGCC-3'	5'-AACCAAGGGAGCTTCAGGGTCA-3'
Cxcl2	5'-CATCCAGAGCTTGAGTGTGACG-3'	5'-GGCTTCAGGGTCAAGGCAAACT-3'
Icam1	5'-CTGGGCTTGGAGACTCAGTG-3'	5'-CCACACTCTCCGGAAACGAA-3'
18S	5'-ACATCCAAGGAAGGCAGCAG-3'	5'-TTTTCGTCACTACCTCCCCG-3'
